# Evaluation of Vibration Damping Enhancement in Laminated Aluminum Sheets for Automotive Application

**DOI:** 10.3390/ma17174421

**Published:** 2024-09-08

**Authors:** Jong-Hwa Hong, Hyeonil Park, Se-Jong Kim, Daeyong Kim

**Affiliations:** 1 Materials Processing Research Division, Korea Institute of Materials Science, 797 Changwondaero, Changwon 51508, Gyeongnam, Republic of Korea; jhong@kims.re.kr; 2 Aerospace Materials Research Center, Korea Institute of Materials Science, 797 Changwondaero, Changwon 51508, Gyeongnam, Republic of Korea; 3 Materials Data & Analysis Research Division, Korea Institute of Materials Science, 797 Changwondaero, Changwon 51508, Gyeongnam, Republic of Korea; 4 Department of Intelligent Mobility, Chonnam National University, 77 Yongbong-ro, Buk-gu, Gwangju 61186, Republic of Korea

**Keywords:** laminated aluminum sheets, vibration analysis, damping ratio, automotive dash panel

## Abstract

In this research, the vibration damping characteristics of the laminated aluminum sheets (LAS) were evaluated in a sheet specimen and an automotive dash panel and compared with those of the monolithic aluminum sheet (MAS). The LAS was fabricated with two 5xxx series aluminum alloy (AA) sheets (AA5052-O) with a thickness of 0.7 mm by inserting an acryl-based adhesive in between. The automotive dash panels were manufactured by multi-step stamping processes for the LAS and the MAS with a similar thickness. The shaker vibration test in a sheet specimen and the impact hammer test in an automotive dash panel were conducted to measure the frequency response function (FRF) of LAS, compared with those of MAS. The results show that the frequency response function made by the LAS has less noise and fluctuation than that of the MAS in a sheet specimen and an automotive dash panel. The damping ratios in a sheet specimen and an automotive dash panel made by the LAS have higher values than those of the MAS. This proves that the LAS has better vibration damping characteristics and a larger damping effect than the MAS in a sheet specimen and an automotive dash panel.

## 1. Introduction

Noise and vibration occupy an important role in the automobile industry for a variety of reasons. Noise and vibration can impair the comfort of drivers and passengers and influence the impression and the overall image of a vehicle while riding. The unexpected fatigue failure of automotive parts due to repeated vibrations will also affect vehicle durability [1]. For this reason, the noise and vibration damping characteristics inducing the problems should be considered important in the process of manufacturing automotive parts [2,3]. Until now, studies on the vibration damping characteristics of various automobile parts have been conducted from the design process. It is necessary to collect data on the noise and vibration sources generated by the vehicle body to identify the noise sources and to reveal their contribution [4,5]. There have been studies on vibration and noise generation due to engine combustion in the powertrain [6,7,8], studies on the analysis of the contribution of each factor through analysis of the driving system in the development stage of the small bus [9], and studies on the noise and vibration of automotive parts such as disc brakes [10,11,12], tires [13,14], and so forth.

After analyzing the noise and vibration of the car body, the results have to be reflected in the automotive design to improve it. There are basic design practices to achieve the reduction of noise and vibration based on the classification of the vibration of the car body and the understanding of the basic mechanism affecting the transmission of noise [15]. For example, concerning the vibration directly transmitted to the vehicle body, efforts should be made to dampen the vibration through the active and semi-active control in the suspension system [16].

Another way to dampen the vibration of a vehicle is to use a viscoelastic material to reach the goal through passive damping [17,18,19]. The damping effect of the viscoelastic material comes from the viscous properties of elastic material where the viscoelastic material is considered as a combination of elastic spring and viscous dashpot. The fundamental theory of viscoelasticity is established based on the arrangement of the elastic spring and viscous dashpot and its number of components [20]. As the spring and dashpot are connected in series, this is called the Maxwell model, which was developed by Maxwell [21]. Due to the independent behavior of the dashpot, the strain infinitely increases in creep stress conditions, such as the presence of fluid. Another simple representative model is the Kelvin model. Compared to the Maxwell model, the Kelvin model shows saturated behavior in the creep stress condition. Both models, however, are too simple to represent highly complex viscoelastic behavior. For this, the general viscoelasticity model expressed with a series or integration form is normally utilized, which is called hereditary integrals.

As a way to use a viscoelastic material, laminated aluminum sheets (LAS) are produced by placing viscoelastic polymeric materials in between the sheets and are normally applied to vibration-affected applications such as automotive parts [22,23,24]. In general, steel sheets have been mainly studied for the laminated sheets for application to automobile parts. There have been studies conducted on the application of damping steel sheets to resistance spot welding, and studies on the formability of damping steel sheets [25,26]. Research conducted on vibration damping characteristics of damping steel sheets has also been steadily conducted [27,28,29,30]. Recently, to keep pace with the problem of weight reduction of the car body, research on aluminum damping sheets has been progressing. Most of the research, however, has focused on the mechanical properties like plasticity and the formability of the laminated (sandwich) materials [31,32,33,34]. The studies on the vibration damping properties of the material itself and those of its application in automotive parts are still insufficient.

In this study, the LAS was manufactured using a rolling process with the 5xxx series aluminum alloy sheets (AA5052-O) with monolithic aluminum sheets (MAS) using the acrylic adhesive in between the layers. The basic material properties of the LAS were compared with those of the MAS. In order to analyze the vibration damping characteristics of the MAS and the LAS, sets of vibration tests were performed in a sheet specimen and an automotive dash panel. To analyze the vibration damping characteristics in an automotive dash panel, prototypes were manufactured through the drawing, the re-striking, the piercing, and the trimming processes, and then, by measuring the damping ratio and the frequency response function (FRF) of the dash panel made of the MAS and of the LAS. In this paper, the vibration damping characteristics of the LAS were evaluated through the above procedure, and the effectiveness of the LAS as a lightweight part was revealed in a sheet specimen and an automotive dash panel.

## 2. Materials

### 2.1. Fabrication of the Laminated Aluminum Sheets (LAS)

**Figure 1:**
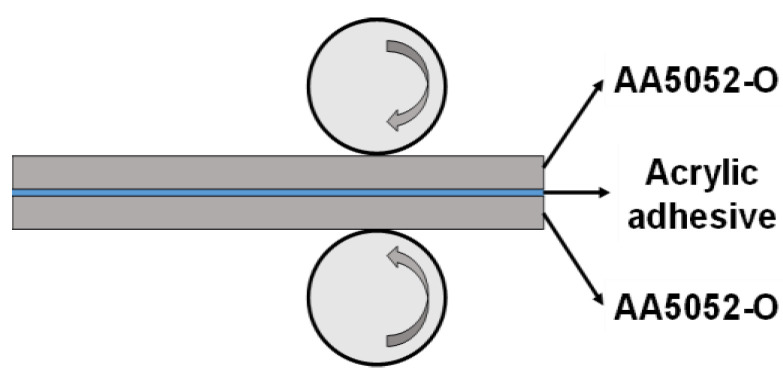
A schematic view of the roll bonding process to produce the LAS.

**Figure 2:**
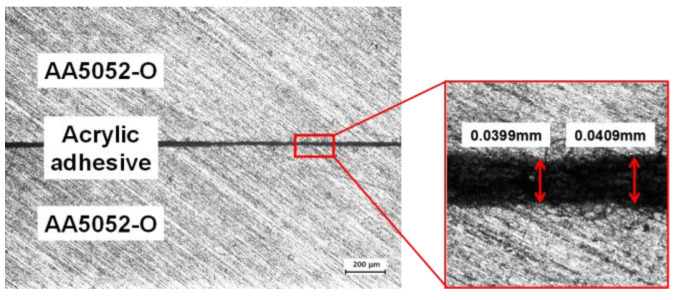
A vertical cross-section of the produced LAS.

In order to manufacture the LAS, two 5xxx series aluminum alloy (AA) sheets (AA5052-O) were utilized as the MASs with a thickness of 0.7 mm. As an adhesive to attach two MASs, an acryl-based adhesive which was determined throughout the prior study for DP580 steel was used [35,36]. Thereafter, conductive particles were inserted into the acrylic adhesive for the application to resistance spot welding. The roll bonding process was used to manufacture the LAS and the schematic view was shown in Figure 1. The vertical cross-section of the produced LAS is shown in Figure 2. There was a difference in the thickness of the adhesive depending on the location, but it was found to be about 0.04 mm on average.

**Figure 3:**
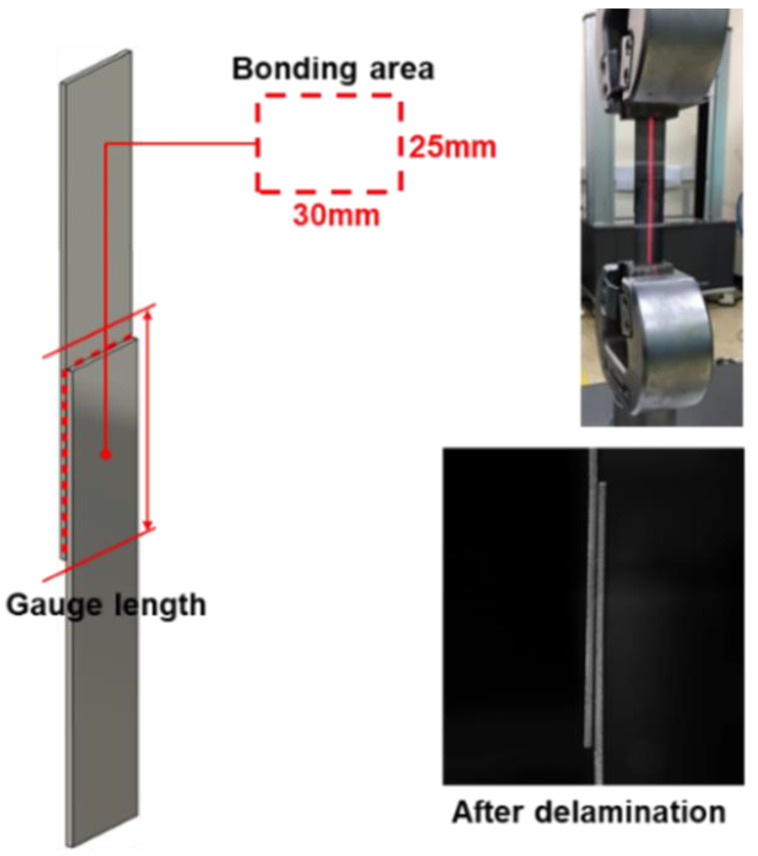
Experimental setup of the single lap joint test and the results after the delamination.

**Figure 4:**
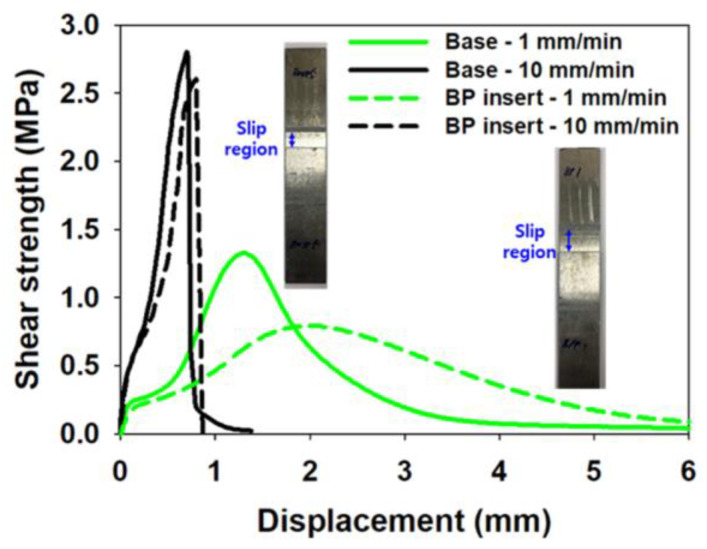
Dependence of lap shear behavior on the stroke rate, as modified by the presence of conductive particles in the acrylic adhesive.

For the performance of the adhesive and the degree of deterioration due to the addition of the conductive particles, a series of experiments were conducted to confirm interfacial slippage. Figure 3 shows the experimental setting for the single lap joint test and the test results after delamination. Based on the ASTM D1002 standard, a specimen for the single lap joint test by applying an acrylic adhesive which has conductive particles in the overlap area of 25 mm × 30 mm was prepared in order to see how the adhesion performance was influenced because of the addition of the conductive particles [37]. Displacement was measured with a laser extensometer with the 40 mm initial gauge length, and the test was performed at stroke speeds of 1 mm/min and 10 mm/min. Due to the viscoelastic properties of the strain-rate sensitive adhesive, interfacial slip occurred rather than adhesive breakage. The stroke rate sensitive lap shear behavior depending on the presence of the conductive particle is shown in Figure 4. There was a slight difference in adhesion behavior depending on the presence of the conductive particles and the difference in behavior became larger as the stroke speed became slower. However, it was thought that the result did not have a significant effect on production during the forming process.

For the measurement of the mechanical properties of the MAS and the LAS, a series of uniaxial tensile tests were performed. The basic mechanical properties of the MAS and the LAS along the rolling direction (RD), diagonal direction (DD), and transverse direction (TD) are summarized in Table 1. The yield stress, the ultimate tensile stress, the uniform elongation, and the total elongation were compared as the representative material properties. The materials show anisotropic behavior depending on the material directions. However, there are no big differences between the MAS and the LAS. Therefore, it is confirmed that the adhesive does not influence the overall mechanical properties.

### 2.2. Automotive Dash Panel with LAS

**Figure 5:**
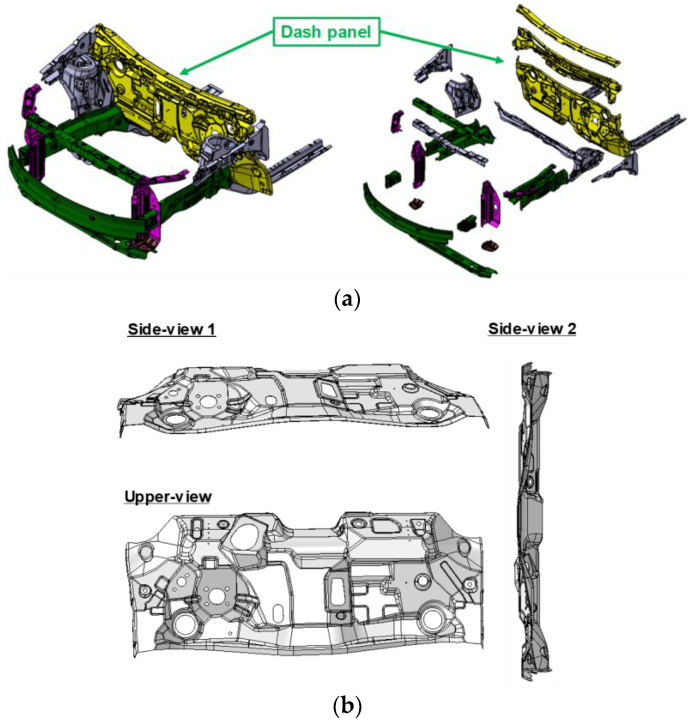
(**a**) The front part of a car’s body structure equipped with a dash panel; (**b**) upper view and two side-views of the optimized geometry of the dash panel.

An automotive dash panel was utilized to investigate the vibration damping characteristics in the automotive application. The front body structure equipped with the dash panel is shown in Figure 5a. The targeted geometry of the dash panel with respect to various views is also shown in Figure 5b. The front body serves to load heavy objects, support the front wheels of the front suspension, and absorb the shock at the same time during an accident without transmitting it to the inside where the passenger stays. Since the front body module of the car body requires lightness, crash-absorbing ability, and structural rigidity, simultaneously, various kinds of materials are utilized in the front body module. The role of the dash panel is to block the noise coming inside, where passengers stay, from the engine room. Therefore, vibration damping sheets were utilized for the passive vibration control in this study.

**Figure 6:**
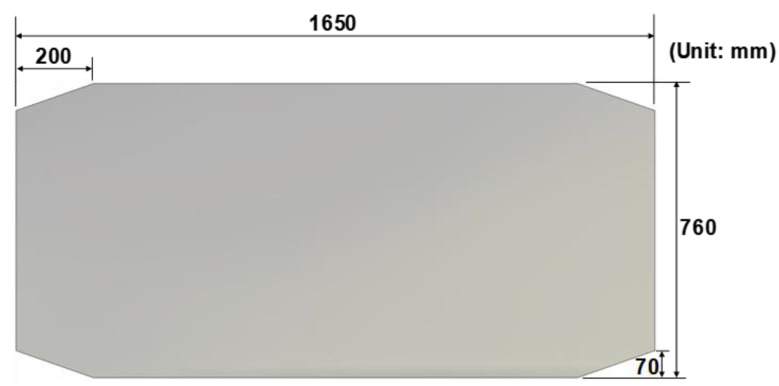
The schematic view of the initial blank.

**Figure 7:**
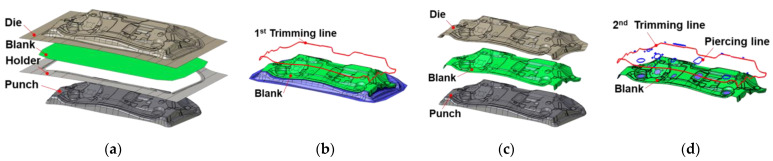
A schematic representation of the five sequential manufacturing processes for the production of the optimized dash panel: (**a**) OP10: drawing; (**b**) OP20: 1st trimming with 3D laser cutting, (**c**) OP30: re-striking, (**d**) OP40 and OP50: 2nd laser trimming and piercing process.

An automotive dash panel was manufactured using the sheet metal stamping process. Figure 6 shows the schematic view of the initial blank. The process sequentially consists of five different operations (OP) as shown in Figure 7: (a) OP10 drawing, (b) OP20 1st trimming with 3D laser cutting, (c) OP30 re-striking, and (d) OP40 2nd laser trimming and OP50 piercing. In OP10, the blank is held by the holder with a die followed by the drawing process by the punch. After the initial blank is drawn, the first trimming and cutting are performed with a laser in OP20. Then, the second forming without the holder, called re-striking, is processed in OP30. The formed blank is trimmed again with the additional piercing process, which makes closed holes on the panel in OP40 and OP50.

## 3. Experiments

### 3.1. Shaker Vibration Test in a Sheet Specimen

**Figure 8:**
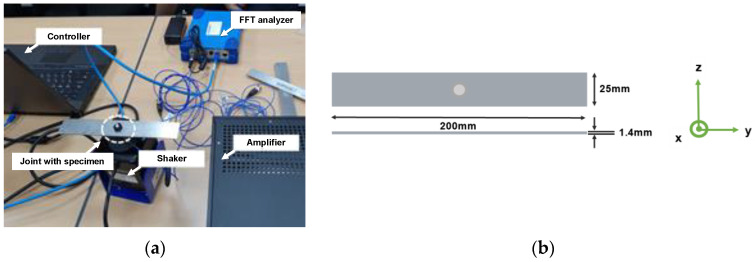
(**a**) Experimental setup of the shaker vibration test with a sheet specimen; (**b**) geometry of the rectangular specimen.

For the vibration damping characteristics and damping effect of the LAS, a vibration test was prepared in a sheet specimen with a shaker vibration test. Figure 8a shows the experimental setting for the vibration test and Figure 8b shows the geometry of the rectangular specimen for the test. As excitation is applied to the specimen from the shaker, vibration information is received through the accelerometer. This information enters an FFT (Fast Fourier Transform) analyzer amplified by the signal amplifier, and vibration information, along with the time, is converted into the information along with frequency. The vibration experiment was attempted for each specimen made of the MAS and the LAS. The FRF was received in the vibration range from 1 Hz to 4000 Hz.

**Figure 9:**
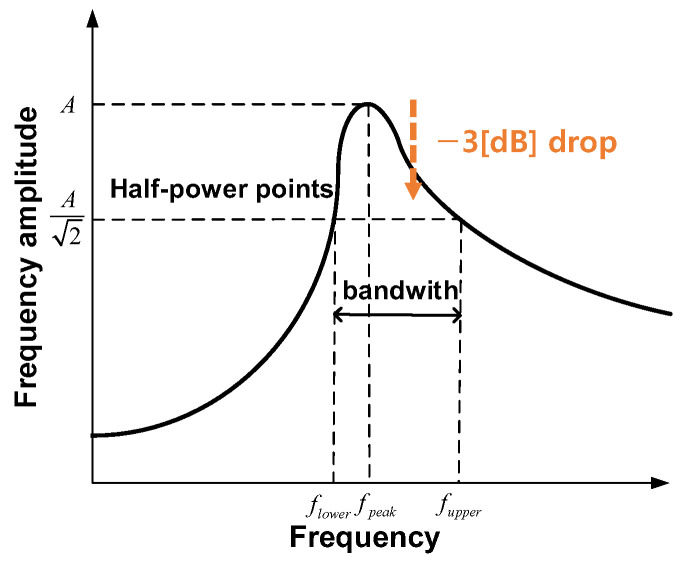
Estimation of damping ratio with the half-power band width method.

The half-power bandwidth method is one of the simplest ways to estimate the damping ratio (ρ) [38]. The method is widely and commonly utilized by vibration engineers in various vibration application fields e.g., architecture, automobile, etc. [39,40]. The half-power bandwidth method can be calculated by using the following equation through the frequencies corresponding to half-power points around the local maximum frequency, as shown in Figure 9.

(1) $$\rho =\frac{\Delta f}{2\cdot f_{peak}}=\frac{f_{upper}-f_{lower}}{2\cdot f_{peak}}$$

where $f_{peak}$ is the local maximum frequency, which is one of the natural or resonance frequencies, and $f_{lower}$ and $f_{upper}$ are the frequencies corresponding to half-power points around the local maximum frequency. The half-power bandwidth method is also called the three dB method [41,42,43]. In the decibel scale, the half-power corresponds to −3 [dB] due to $20\log_{10}(\frac{1}{\sqrt{2}})\approx -3\left[\mathrm{dB}\right]$. Therefore, the three dB method is utilized in the study in the decibel scale amplitude.

### 3.2. Impact Hammer Test in an Automotive Dash Panel

**Figure 10:**
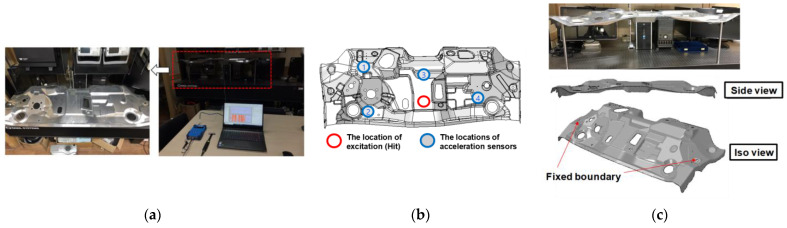
(**a**) Experimental setup for the impact hammer test with the dash panel; (**b**) positions of the sensor attachment and the impact region: P1, P2, P3, and P4; (**c**) fixed boundary conditions of the dash panel.

By using the final product of the automotive dash panel, the impact hammer tests were also conducted on the dash panel. Two dash panels made of the LAS and the MAS were utilized for comparison. The experimental setup for the vibration characteristics is shown in Figure 10a. For FFT (Fast Fourier Transform) analysis, the eight (8)-channels Mobile DAQ System (OR10) was used, and the test was performed by using an impact hammer (086C03) and six acceleration sensors (352C33). After the signal obtained from excitation by an impact hammer was amplified with the signal amplifier, the FRF was obtained throughout the experiment. Figure 10b shows the positions of the four attached acceleration sensors and the hammer impact region. The accelerometers utilized in this study vertically move along the uniaxial direction (*z*-axis) and the sensors were attached to the blue circle position. An excitation location by the impact hammer is in the red circle position. The dash panel was fastened by the bolts at the pierced holes. The fixed boundary conditions of the dash panel for the experiment are shown in Figure 10c.

In general, automobile noise is divided into air-borne noise and structure-borne noise [44]. Air-borne noise mainly occurs at high frequencies above 500 Hz, but structure-borne noise mainly occurs at low frequencies below 200 Hz. For the test in an automotive dash panel, frequency ranges up to 150 Hz were considered. The human body commonly feels tired and gets fatigued in a vibration frequency less than 50 Hz and the engine rotation speed is mainly 1000 rpm to 3000 rpm during driving. In the case of a six-cylinder engine, as the rotational speed ranges between 1000 rpm and 3000 rpm can be converted into the frequency, this becomes 50 Hz to 150 Hz. Since this can be viewed as the main vibration source of the vehicle, unless there are few external vibration factors, such as when driving on a smooth road, the study was focused on the frequency ranges up to 150 Hz.

## 4. Results and Discussion

### 4.1. Vibration Characteristics in a Sheet Specimen

**Figure 11:**
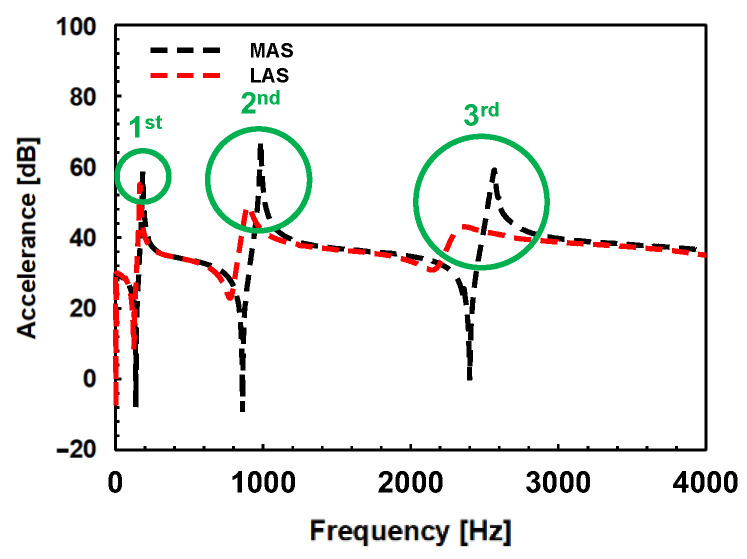
The FRF and the major peaks of the MAS and the LAS and comparison of major peaks.

Figure 11 shows the FRF for the MAS and the LAS. The vertical axis is the accelerance whose acceleration value is divided by unit force. The accelerance can be converted into the unit of decibels using the following conventional logarithm.

(2) $$\mathrm{Accelerance}\left[\mathrm{dB}\right]=20\cdot \log_{10}(\mathrm{Acceleration}/\mathrm{Force}).$$

The frequencies of each peak of the LAS were slightly smaller than those of the MAS. This is because the thicknesses of the MAS and the LAS are not exactly the same. In general, the thinner the thickness, the shorter the period, and the closer the interval between the natural frequencies. In the case of the LAS used in this study, two aluminum alloy sheets with a thickness of 0.7 mm were joined by an adhesive to have a thickness of approximately 1.44 mm, but the MAS has a thickness of about 1.5 mm. The measured frequency, the accelerance peak, and the measured damping ratio of each specimen are shown in Table 2. The damping ratios of the MAS and the LAS were compared by percent change using the following formulation:

(3) $$\delta \left(\%\right)=\frac{\left|\rho_{L}-\rho_{S}\right|}{\rho_{S}}\times 100\left(\%\right)$$

where, $\rho_{S}$ is the damping ratio of the MAS, and $\rho_{L}$ is that of the LAS. The measured damping ratios were slightly different depending on the major peaks. At the first major peaks, the damping ratios were 0.0405 for the MAS and 0.0448 and the LAS, respectively. The percent change in the damping ratio was about 10.45%. At the second major peak, the damping ratio of the LAS is 0.0348, which is 357.29% higher than the damping ratio of the MAS, which is 0.0079. At the third major peak, the damping ratio of the MAS, which is 0.0058, was increased to 0.0836 of the LAS’s damping ratio value, whose percent change is 1329.52%. The percent change of the damping ratio increased from a minimum of 10.45% to a maximum of 1329.52%. The damping ratio does not differ significantly at the first major peaks. However, as the peak progresses to a higher frequency, the damping ratio deviation between the two materials increases. At each major peak, the amplitude of the accelerance peak of the LAS was also less than that of the MAS in the entire frequency range. Therefore, it can be concluded that the damping properties of the LAS are improved in a sheet specimen from the perspective of the damping ratio as well as the level of FRFs. 

**Figure 12:**
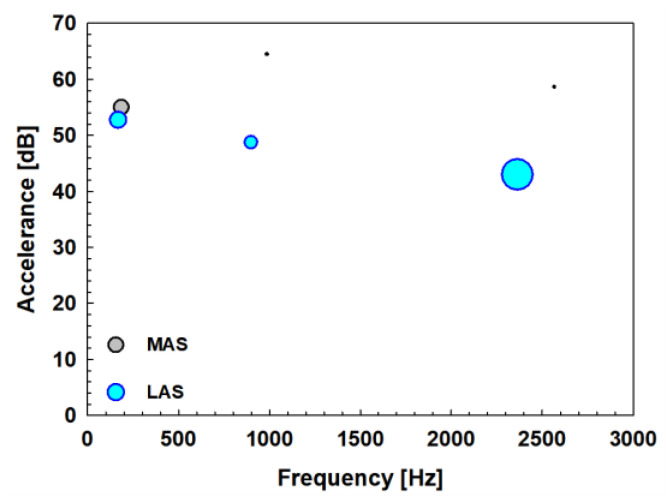
Summary of the major peaks obtained from the FRFs made of the MAS (gray circle) and the LAS (cyan circle) in a sheet specimen.

The frequency, amplitude, and damping ratio of the peaks in a sheet specimen are differently presented in Figure 12. The circles are located at the major peak frequency (*x*-axis) and the peak amplitude (*y*-axis). The MAS is colored gray, and the LAS is colored cyan. The size of the circle represents the damping ratio of each major peak. The larger size of the circle means that the damping ratio is relatively large, and the size is not the absolute value of the damping ratio. In the lower frequency, there is not much difference in the size and height of the gray circle and the cyan circle. However, as the frequency increases, it can be seen that the height of the gray circle is kept high, while the height of the cyan circle is gradually decreased. In addition, it can be seen that the size of the gray circle is gradually reduced, while the size of the cyan circle is constantly maintained large. In other words, in the case of a sheet specimen made of the MAS, it is more vulnerable to noise and vibration at the overall frequency than that made of the LAS. On the other hand, in the case of the dash panel made of the LAS, the peak amplitude is gradually lowered even at high frequencies, and it can be seen that the performance of the damping ratio does not drop at the overall frequency. Therefore, it can be concluded that the vibration damping characteristics of the LAS are superior to those of the MAS in terms of the peak amplitude and damping ratio in a sheet specimen.

### 4.2. Vibration Characteristics in an Automotive Dash Panel

**Figure 13:**
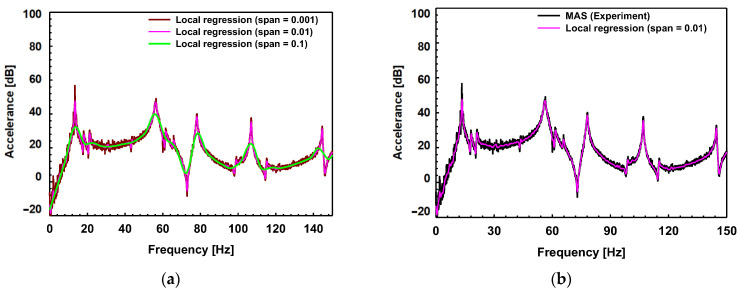
(**a**) Sensitivity test of span value; (**b**) comparison of the regressed curve with experimental raw data (FRF at P2 in Figure 10b as an example).

The FRFs were obtained from each sensor attached to the surface. The impact hammer test in an automotive dash panel has much more noise on its FRFs compared to the shaker test in a sheet specimen. The noise makes it difficult to analyze the FRFs from an automotive dash panel. Before analyzing the FRFs, the local regression with the 2nd-order polynomial model was utilized. If the span value is small, there is less effect on erasing noise. If the higher span value is used, however, the shape of the graph becomes distorted from the original FRFs. Hence, the sensitivity of the span value was investigated as shown in Figure 13a. The value of 0.01 was chosen as the local regression span and compared with experimental raw data as shown in Figure 13b.

To analyze and compare the vibration damping characteristics between the two materials, the major peaks were established. Note that the major peak is related to the natural frequency but is not the exact natural frequency. The major peak is defined by the authors to compare the vibration damping characteristics of the MAS and the LAS. The points considered comparable points were set as the standard of the major peak.

**Figure 14:**
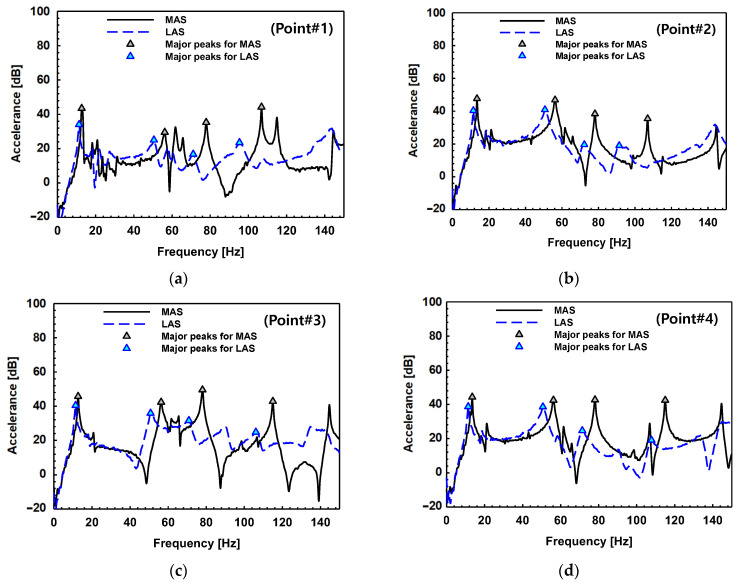
Frequency response function (FRF) of the dash panel made of the MAS and the LAS for each sensor at: (**a**) P1; (**b**) P2; (**c**) P3; (**d**) P4.

The FRFs of the dash panel made of the MAS and the LAS are shown in Figure 14 for each location: (a) Point#1 (P1), (b) Point#2 (P2), (c) Point#3 (P3), and (d) Point#4 (P4). To explicitly compare the difference between the two materials, the FRFs for each material were drawn together. The experiments were performed three times and one of the tests was shown as representative. There is a tendency where the fluctuation of the FRFs decreases as the frequency increases for the LAS because of the damping effect and the phenomenon getting bigger. The measured frequency, the peak amplitude, and the measured damping ratio of the MAS and the LAS are arranged in Table 3 with respect to the number of points. The percent changes in the damping ratio between the dash panel made of the MAS and that made of the LAS are also calculated using Equation (3). The table shows that all measured damping ratios of the LAS dash panel are higher than damping ratios of the MAS and there is no exception. Specifically, the damping ratios in an automotive dash panel made by the LAS have a higher value, with a minimum of 29.41% and a maximum of 1369.59%, than those of the MAS.

**Figure 15:**
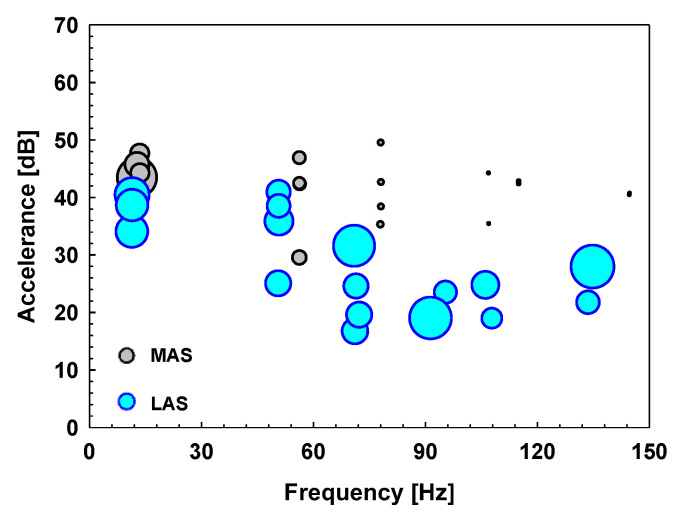
Summary of the major peaks obtained from the FRFs made of the MAS (gray circle) and the LAS (cyan circle) in an automotive dash panel.

The frequency, amplitude, and damping ratio of the peaks in the dash panel are shown in Figure 15 as presented in the previous section. The overall trend is the same as that in a sheet specimen. As the frequency increases, the height of the gray circle is kept high, while the height of the cyan circle is decreased. In addition, the size of the gray circle becomes reduced, while the size of the cyan circle remains relatively large, showing that a dash panel made of the MAS is more vulnerable to noise and vibration than that made of the LAS at the overall frequency, concluding that the vibration damping characteristics of the LAS are superior to those of the MAS concerning the peak amplitude and damping ratio in the dash panel.

**Figure 16:**
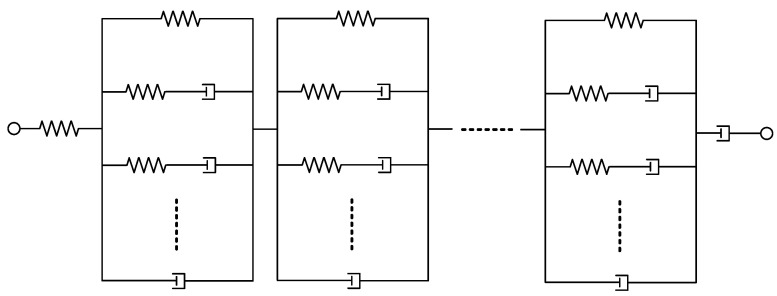
A generalized viscoelastic model arranged sequentially.

Viscoelastic damping is a common mechanism that combines both elastic and viscous behaviors. Viscoelastic materials exhibit properties of both solids (elastic) and liquids (viscous). They store part of the vibration energy generated under a load, while the remaining energy is dissipated as heat returning less energy than its absorption. A generalized viscoelastic model arranged sequentially is shown in Figure 16. The system comprises viscoelastic elements, such as springs (elastic components) and dashpots (viscous components), connected in series. This setup captures both the elastic response (instantaneous recovery of shape) and viscous response (time-dependent deformation) of a material under a load. Depending on the model, it is used to predict the material’s behavior under various loading conditions, accounting for both immediate and delayed deformation. The related research is planned in the future work.

## 5. Summary

The LAS was manufactured using a rolling process with the 5xxx series aluminum alloy sheets (AA5052-O) as the MAS attached to the acrylic adhesive. For the application of welding, the conductive particles were inserted into the adhesive, not much influencing the performance of viscoelastic properties. Afterward, the material properties of the LAS sheets were compared with those of the MAS sheets, and then several vibration tests were conducted in a sheet specimen and an automotive dash panel in order to reveal the improved vibration damping characteristics of the LAS. This research can be applied to the automotive industry to help produce car bodies that minimize driver vibrations by using various joining methods, such as welding during assembly, with the results below:

- The yield stress, the ultimate tensile stress, the uniform elongation, and the total elongation of the LAS were compared with those of the MAS as the representative material properties. However, the material properties do not show big differences between the two materials.
- For the vibration damping characteristics in a sheet specimen and an automotive dash panel, the automotive dash panel was manufactured with five sequential operations consisting of the draw, the restrike, the piercing, and the trimming process. The overall vibration was reduced in the frequency response function in terms of peak amplitude and damping ratios for the LAS in a sheet specimen and an automotive dash panel. It was confirmed that the LAS has a better noise and vibration damping effect compared to the MAS.
- Based on the definition of the half-power bandwidth method, the calculated damping ratio is proportional to the difference in frequency. As the thickness increases, the frequency increases in width. Hence, thicker material (the MAS) normally shows a higher damping ratio value obtained from the half-power bandwidth method. Although the thickness of the LAS is a little thinner than that of the MAS, the damping ratio is consistently small in the study. The works conclude that the vibration damping characteristics of the LAS are superior to those of the MAS in a sheet specimen and an automotive dash panel. Specifically, the percent change in the damping ratio of a sheet specimen increased from a minimum of 10.45% to a maximum of 1329.52%. In contrast, an automotive dash panel shows the percent change in damping ratios, ranging from a minimum of 29.41% to a maximum of 1369.59%.

## Figures and Tables

**Table 1:** Estimated mechanical properties of the MAS and the LAS.

Materials	Direction	Yield Stress (MPa)	Ultimate Tensile Stress (MPa)	Uniform Elongation (%)	Total Elongation (%)
MAS	RD	95.8	204.2	16.5	19.7
	DD	94.9	198.2	20.2	27.1
	TD	96.5	195.7	21.3	25.3
LAS	RD	94.8	202.6	18.9	20.3
	DD	93.9	195.9	25.3	27.3
	TD	93.9	194.3	22.1	25.3

**Table 2:** Estimated frequency response properties of the MAS- and the LAS-made parts.

Major Peaks	Frequency (Hz)		Peak Amplitude (dB)		Damping Ratio		*δ* (%)
	MAS	LAS	MAS	LAS	MAS	LAS	
1st	185.00	167.50	55.04	52.78	0.0405	0.0448	10.45
2nd	985.00	897.50	64.51	48.80	0.0076	0.0348	357.29
3rd	2565.00	2362.50	58.66	43.05	0.0058	0.0836	1329.52

**Table 3:** Measured frequency, peak amplitude, and damping ratio of each material type by the four sensor points in an automotive dash panel.

Major Peaks	Frequency (Hz)		Peak Amplitude (dB)		Damping Ratio		*δ* (%)
	MAS	LAS	MAS	LAS	MAS	LAS	
P1							
1st	12.70	11.33	43.47	34.08	0.0346	0.0448	29.41
2nd	56.20	50.54	29.54	25.07	0.0122	0.0222	81.97
3rd	77.93	71.09	35.33	16.76	0.0053	0.0227	326.27
4th	106.89	95.36	44.26	23.52	0.0023	0.0197	762.98
P2							
1st	13.43	11.43	47.69	40.4	0.0164	0.0278	69.51
2nd	56.2	50.63	46.91	40.96	0.0109	0.0207	89.91
3rd	78.08	72.22	38.43	19.61	0.0050	0.0223	345.7
4th	106.93	91.36	35.45	19.03	0.0025	0.0369	1369.59
P3							
1st	12.7	11.38	45.72	40.47	0.0212	0.03	41.51
2nd	56.3	50.73	42.35	35.9	0.0108	0.025	131.48
3rd	78.03	70.85	49.54	31.61	0.0050	0.0362	623.05
4th	114.99	106.06	42.83	24.79	0.0030	0.0239	704.77
P4							
1st	13.48	11.38	44.28	38.68	0.0163	0.0279	71.17
2nd	56.2	50.63	42.48	38.54	0.0104	0.0203	95.19
3rd	78.08	71.39	42.68	24.58	0.0050	0.0215	329.71
4th	114.99	107.81	42.42	18.99	0.0030	0.0177	495.12

## Data Availability

The original contributions presented in the study are included in the article, further inquiries can be directed to the corresponding author.
